# An Investigation of the Feasibility and Acceptability of Using a Commercial DASH (Dietary Approaches to Stop Hypertension) App in People With High Blood Pressure: Mixed Methods Study

**DOI:** 10.2196/60037

**Published:** 2024-11-19

**Authors:** Ghadah Alnooh, Jozaa Z AlTamimi, Elizabeth A Williams, Mark S Hawley

**Affiliations:** 1 Centre for Assistive Technology and Connected Healthcare, School of Medicine and Population Health, University of Sheffield Sheffield United Kingdom; 2 Department of Health Sciences, College of Health and Rehabilitation Sciences, Princess Nourah Bint Abdulrahman University Riyadh Saudi Arabia; 3 Department of Sports Health, College of Sports Sciences & Physical Activity, Princess Nourah Bint Abdulrahman University Riyadh Saudi Arabia; 4 Healthy Lifespan Institute, University of Sheffield. Sheffield United Kingdom

**Keywords:** hypertension, blood pressure, Dietary Approaches to Stop Hypertension, DASH diet, self-efficacy, mobile health, mHealth, Saudi Arabia, mobile phone

## Abstract

**Background:**

The use of smartphone apps for dietary self-management among patients with high blood pressure is becoming increasingly common. Few commercially available DASH (Dietary Approaches to Stop Hypertension) diet apps have the potential to be effective, and only a few of these have adequate security and privacy measures. In previous studies, we identified 2 high-quality apps that are likely effective and safe. One of these, the Noom app, was selected as the most suitable app for use in the Saudi Arabian context based on health care professionals’ and patients’ preferences.

**Objective:**

This study aims to determine the feasibility and acceptability of using the Noom app to support DASH diet self-management among people with high blood pressure in Saudi Arabia.

**Methods:**

This mixed methods study evaluated the feasibility and acceptability of using the Noom app among people with high blood pressure in Riyadh, Saudi Arabia. Fourteen participants with high blood pressure were recruited and asked to use the app for 8 weeks. The quantitative outcome measures were DASH diet adherence and self-efficacy. Feasibility and acceptability were assessed during and after the intervention via the Noom diet-tracking engagement questionnaire, the System Usability Scale, and semistructured interviews.

**Results:**

Most participants (8/13, 62%) logged their meals for 3 to 5 days a week; the frequency of logging increased over time. Snacks were the foods they most often forgot to log. The interviews revealed four main themes: (1) acceptance, (2) app usability, (3) technical issues, and (4) suggestions for improvement. Most participants found the Noom app acceptable, and most had no difficulties integrating it into their daily routines. The results of this feasibility study provided insights into the app’s educational content, some of which was deemed unsuitable for Saudi Arabian users. App usability was identified as a critical theme: the app and its database were easy to use, convenient, and valuable to most of the participants. Despite this, some of the participants reported difficulties in identifying some foods because of a lack of local options on the app. Technical issues included the app freezing or responding slowly. Most participants also suggested developing an Arabic version of the app and simplifying the method of food logging. The participants showed some improvement in self-efficacy and adherence to the DASH diet, although these improvements were not statistically significant. The mean self-efficacy score increased from 18 (SD 4.7) to 20 (SD 6.3), and the mean DASH diet score increased from 3.4 (SD 1.4) to 4.3 (SD 1.1).

**Conclusions:**

The app was feasible and acceptable among the participants who completed the study. Further studies are needed to examine the potential of smartphone apps in promoting adherence to the DASH diet and their impact on blood pressure among individuals with hypertension in Saudi Arabia.

## Introduction

### Background

High blood pressure (hypertension) is the most preventable cause of cardiovascular disease (CVD) and all-cause mortality around the globe [1], affecting 1.28 billion people worldwide [2]. Lifestyle risk factors for hypertension include unhealthy diets, tobacco use, and a lack of physical activity [2]. Hypertension is a significant public health concern in Saudi Arabia and contributes significantly to mortality rates [3,4]. The Saudi Ministry of Health reported in 2023 that 2 out of 5 Middle Eastern adults have hypertension [5]. A recent systematic review and meta-analysis evaluating the prevalence and awareness of hypertension in Saudi Arabia revealed that 23% of Saudi Arabian adults have hypertension but that more than half of them (57.2%) were unaware of their condition and, as a result, did not seek treatment [4]. Undiagnosed, untreated, or uncontrolled hypertension can lead to severe health consequences, such as heart failure, coronary heart disease, and renal failure [2]. These diseases burden the health care system, and mortality rates related to hypertension-induced CVD are expected to rise [6].

The DASH (Dietary Approaches to Stop Hypertension) diet is an effective blood pressure–lowering plan [7-9]. The DASH diet encourages the consumption of fruits, vegetables, whole grains, nuts, lean meat, fish, and dairy products and limited consumption of sodium, saturated fat, sugar-sweetened beverages, and sweets [10,11]. A low consumption of fruits and vegetables and a high consumption of fast foods containing saturated fats, high energy density, and sodium has been associated with obesity and elevated blood pressure in adults and children in Saudi Arabia [12-14]. These data show that addressing the dietary habits of Saudi Arabians may help reduce a significant risk factor for prevalent CVD and hypertension within this population [12-17].

Adherence to the multiple components of the DASH diet plan can be difficult [18]. Self-efficacy is 1 of the core concepts of social cognitive theory and describes people’s confidence in their ability to engage in specific behaviors or achieve desired outcomes [19]. Increasing self-efficacy for healthy eating has been associated with positive behaviors, including increased fruit and vegetable consumption [20]. Self-monitoring is a technique for raising awareness of an individual’s behavior through recording details about the behavior performed [20,21]. By receiving feedback on self-monitoring behaviors, such as calories consumed daily, one may be able to change one’s behavior (eg, reducing caloric intake) to achieve the desired results.

Mobile phone apps have been shown to improve nutritional habits and medical care [22]. Using apps to improve diet and nutrition can be a valuable and low-cost intervention strategy [22]. Dietary smartphone apps can estimate changes in eating behavior (eg, self-reported food, energy, micro- and macronutrient, and fruit and vegetable intake) and outcomes (eg, body weight, blood pressure, blood glucose levels, physical activity, and quality of life) [22].

As part of Saudi Arabia’s Vision 2030 program, there is a push to leverage mobile health apps in the health care sector to enhance patient care [23]. The Saudi Ministry of Health has introduced multiple mobile apps, such as the Sehhaty (Arabic for “health”) app, to streamline administrative procedures for users [24]. This app provides convenient access to medical consultations; enables users to update their medication information; and allows for the monitoring of vital health indicators such as blood pressure, BMI, waist circumference, and blood glucose levels [25]. According to Alrowaily et al [26], primary health care physicians are taking a leading role in the adoption of telehealth by increasingly using the Sehhaty mobile app, thereby influencing the general population’s acceptance of this technology. In addition, several studies reported that Saudi users accept app-based interventions, and diet-tracking apps are convenient and more accessible than other tracking methods [27-29].

A systematic review assessing the effectiveness of smartphone apps to support DASH diet self-management found weak emerging evidence of a positive effect of apps in enhancing dietary self-management [30]. A recent systematic search of commercial app stores and a content analysis of DASH diet apps found that only a limited number of high-quality apps support DASH diet self-management, and only 2 apps (Noom and DASH To Ten) were deemed to be high quality and potentially effective and to offer adequate security and privacy protection [31]. A subsequent study (unpublished), conducted in Saudi Arabia, explored family physicians’, dietitians’, and patients’ perceptions of the 2 apps, confirming their potential effectiveness, high quality, and good security and privacy measures. When the participants were asked to select their preferred app, the Noom app was considered the most suitable. Health care professionals and patients widely accepted Noom because it has interaction-enabled functions, provides a comprehensive food database that helps patients track their food, and offers mindful eating strategies.

The Noom smartphone app promotes healthy behavior change [32,33]. This app includes features aligned with social cognitive theory, such as setting manageable goals, monitoring daily progress, receiving feedback, participating in social support, and problem-solving [31,33-35]. A broad range of behavioral strategies are included in the Noom program, such as promoting a healthy lifestyle and suggesting food choices [34]. Furthermore, published evidence from a study conducted in the United States supports the usability and effectiveness of the Noom app to reduce weight and high blood pressure [36]; however, it is important to note that the study had a small sample size and only focused on individuals aged 40 to 50 years.

Before evaluating the effectiveness of an app in a new setting, it is critical to examine its feasibility and acceptability among end users and their willingness to use it [37,38]. This step is essential because systems that are difficult to use may yield low goal-achievement efficiency or result in users neglecting or rejecting the technology [38]. Considering the chosen context of this study, the research investigates the feasibility and acceptability of the adopted Noom app to ensure that it is well suited to a new context, specifically for people with high blood pressure in Saudi Arabia.

### Study Aim

This study aims to determine the feasibility and acceptability of using the Noom app to support DASH diet self-management among people with high blood pressure in Saudi Arabia.

### Objectives

The objectives of the study were to determine the usability of the app, adherence to the DASH diet, and self-efficacy, as well as to investigate the patient experience and satisfaction with the app (including ease of use and any technical problems encountered).

### Research Question

We developed the following research question: is it feasible and acceptable to use the Noom app to assist people with high blood pressure to improve their dietary habits?

## Methods

### Research Design

In this study, a 1-group pretest-posttest design was adopted to investigate the feasibility of an 8-week intervention to determine whether patients with high blood pressure living in Saudi Arabia can use the Noom app to manage their diets. A mixed methods approach was used. Adherence to the DASH diet and self-efficacy were evaluated using objective quantitative measures, while both quantitative and qualitative methods were used to assess patient satisfaction with the Noom app. The CONSORT (Consolidated Standards of Reporting Trials) guidelines for feasibility trials were followed to report this study [39] (Multimedia Appendix 1).

### Study Setting

All participants were enrolled and followed remotely for 8 weeks.

### Participants

Patients with high blood pressure attending outpatient clinics at the King Abdullah bin Abdulaziz University Hospital in Riyadh, Saudi Arabia, who had participated in an earlier qualitative study (unpublished) and who had agreed to be contacted for future research were sent an electronic message through WhatsApp inviting them to take part in this study. The earlier study received approval from the School of Health and Related Research of the University of Sheffield (049904), Princess Nourah bint Abdulrahman University (22-0490), and the King Abdullah bin Abdulaziz University Hospital (22-0054). The clinical team (nurses) and primary researchers identified eligible patients for the earlier study during regular clinic appointments. The inclusion criteria were as follows: individuals who (1) had systolic blood pressure ranging from 130 mm Hg to 159 mm Hg and diastolic blood pressure ranging from 85 mm Hg to 99 mm Hg [40], (2) were aged ≥18 years, (3) had a BMI of ≥25 kg/m^2^, (4) were willing to read content in English because the Noom app is not available in Arabic, and (5) owned a smartphone and were willing to receive monthly SMS text messages. Individuals who (1) had systolic blood pressure of ≥160 mm Hg and diastolic blood pressure of ≥100 mm Hg [40], (2) had CVD, (3) had a history of renal diseases, or (4) were pregnant or planning to become pregnant while the study was ongoing were excluded from the study. Twelve participants were considered an adequate sample size for this feasibility study [41]. The purpose of this study was not to detect effects using inferential statistical tests; therefore, power calculations were not performed [42].

### Study Procedure

**Figure 1:**
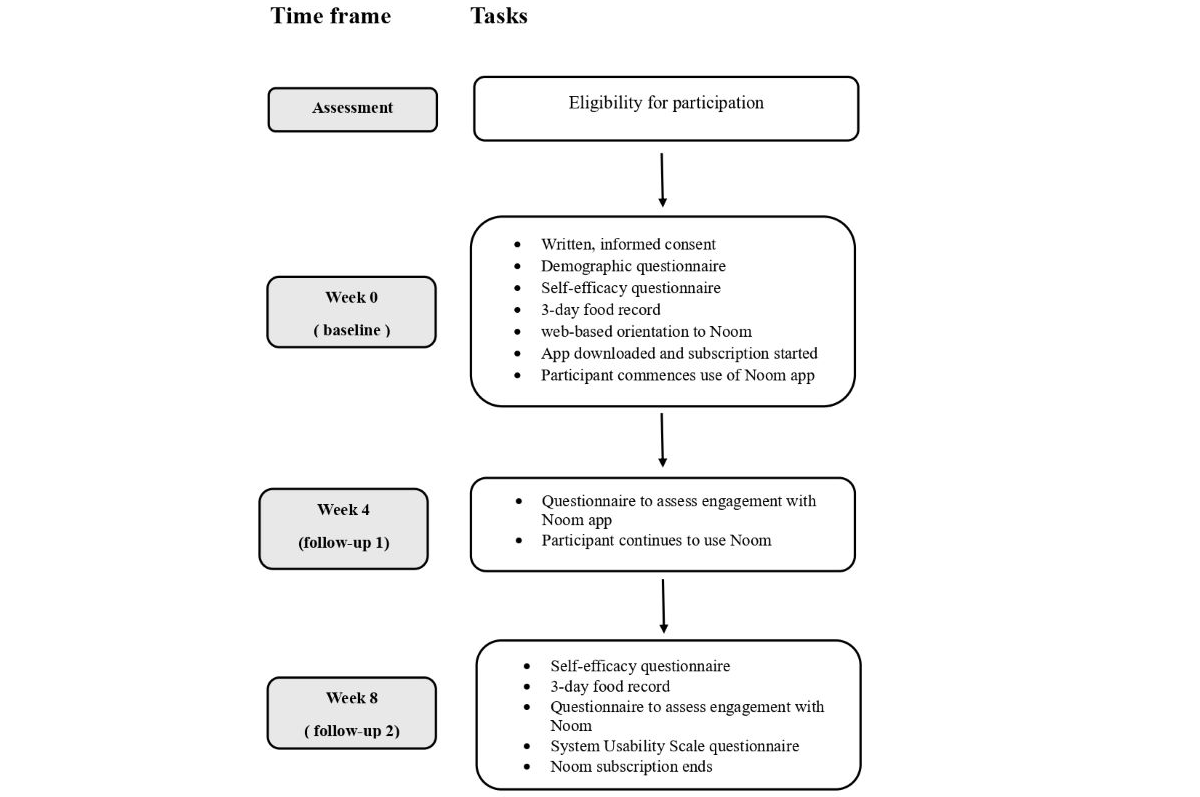
An overview of the time frame of the study and the tasks involved in the study protocol. SUS: System Usability Scale.

Figure 1 summarizes the study procedure. Eligible individuals were asked to review and sign a consent form sent via email. They then completed a self-reported demographic survey, a self-efficacy questionnaire, and a 3-day food record. Subsequently, in the first web-based orientation session, they were instructed to download and purchase the Noom app (each participant received the equivalent of US $140 [as of June 2023] to cover the cost). The DASH diet and various app functions and features were reviewed with the participants, and they were asked to use the app daily for 8 weeks and to use it to enter all the food and beverages they consumed. After 4 weeks, participants were sent an email asking them to complete the Noom diet-tracking engagement questionnaire. At the end of the intervention, all participants were sent an email asking them to complete questionnaires, including those on Noom diet-tracking engagement, self-efficacy, a 3-day food record, and the System Usability Scale (SUS). Finally, we conducted qualitative, web-based semistructured one-on-one interviews with the participants to gain insights into their experiences and the acceptability of using the app. The interviews also investigated the perceived impact of the app on DASH diet adherence and identified any technical problems encountered in using the app.

### Description of the Noom App

The Noom app has the following features: (1) a tool to track dietary intake using comprehensive food databases and an intelligent recognition system to identify food barcodes (Noom has its own food database, and it uses reliable government sources, such as the US Department of Agriculture’s food database, or directly consults the nutrition labels of packaged foods to provide app users with accurate information [43]); (2) a daily log to record blood pressure, weight, and blood glucose levels, while exercise can be recorded manually or automatically by syncing with a Fitbit device; (3) feedback on the diet and activities recorded; (4) a daily reminder to log diet and other activities, such as physical activity and reading daily articles; and (5) a social communication component that allows participants to communicate with others to share their experience (they can also communicate with a human coach via in-app messages).

Furthermore, the app offers comprehensive content written by nutritionists, physicians, and psychologists to assist users in achieving and maintaining a healthy lifestyle. With a focus on developing healthy eating habits, increasing physical activity, and building skills for overcoming obstacles, the courses cover various topics, including calorie balance, staying active, overcoming unhealthy behaviors, and seeking support from family and friends. In addition, the app emphasizes the importance of regular eating, getting enough sleep, and managing stress. All this content is delivered in short, easy-to-understand segments daily to help users achieve their goals.

### Measures

#### Baseline Assessment

This study measured various demographic variables. Standard survey questions used in the previous study were used to collect demographic measures at baseline [27]. This included age, sex, marital status, educational achievement, and whether participants had ever used a dietary smartphone app outside of the research. Participants were asked to self-report their height and weight, from which BMI was calculated using the following formula [44]: weight (kg) / (height [m])^2^.

#### Quantitative Outcomes

The quantitative outcomes of this study were DASH diet adherence and participants’ self-efficacy, which were assessed at baseline and at the end of the intervention.

To evaluate the change in DASH diet adherence, macro- and micronutrient intakes were assessed using a 3-day food record before and after the intervention. Nutritics dietary analysis software (version 5.95) was used to analyze reported intake to determine the changes in adherence to the DASH diet [45]. Participants were asked via email to complete a 3-day food record before week 1 and at the end of week 8. We calculated a nutrition-based DASH index score, similar to the DASH index score formulated by Mellen et al [46]. The score for DASH diet adherence was computed based on 9 target nutrients: total fat, saturated fat, protein, fiber, magnesium, calcium, sodium, potassium, and cholesterol. A value of 1 was assigned to participants who met the DASH target for a nutrient, 0.5 if they met the intermediate target, and 0 if neither target was met. Thus, the range of scores was 0 to 9, with higher scores indicating greater adherence, while a score of 9 represented full adherence [47].

To measure DASH diet self-efficacy, this study adopted the 6-item diet self-efficacy scale with participants rating each item on a 5-point Likert scale ranging from 1=*not confident at all* to 5=*completely confident*. Total scores for the scale range from 6 to 30, with a higher score indicating greater self-efficacy.

#### Feasibility and Acceptability

##### Overview

Feasibility was indicated by adherence to the DASH diet and engagement with the Noom app. Acceptability was indicated by assessing participants’ satisfaction and their perception of the app’s usability, measured using the SUS.

We could not automatically track user engagement; therefore, we assessed it by asking participants to respond to the Noom diet-tracking questionnaire, which comprised 5 questions about the frequency and timing of logging, the number of days logged, and any missed meals. These questions were adapted from previous studies that evaluated engagement with dietary smartphone apps [48,49].

Participants completed the SUS for usability testing of the app [50]. For nearly 30 years, the SUS has been widely used to measure usability in commercial and research projects (including mobile apps) [51]. The participants were asked to rank the statements on a 5-point Likert scale ranging from 1=*strongly disagree* to 5=*strongly agree*. The SUS provides a final score ranging from 0 to 100, with higher values indicating better usability. The acceptability ranges classify a product’s SUS score to determine whether it has an *acceptable* SUS score or needs more attention and continuous improvement; for example, a score is considered *not acceptable* if it falls between 0 and 50, *low marginal and high marginal* between 51 and 69, and *acceptable* at ≥70 [52].

##### Qualitative Measures: Semistructured Interviews

Feasibility and acceptability were also evaluated using one-on-one semistructured web-based interviews using Google Meet. The interview guide was developed based on previous studies [53,54] and was informed by social cognitive theory [21]. It included detailed questions about participants’ experiences with the Noom app for dietary self-management, as well as questions about the benefits and barriers they encountered when using the app. The interview explored factors influencing app engagement, such as self-monitoring, feedback, goal setting, and motivational strategy. The interview guide (Multimedia Appendix 2) was thoroughly tested with a single volunteer to ensure clarity for the participants. The interviews were conducted on the web because web-based meetings, unlike traditional methods, provide greater flexibility and allow participants to schedule the meeting around their busy schedules and personal commitments [55]. To minimize bias, the interviewer clarified to the participants that they could express their views freely without pressure during the interview. In addition, audio recordings and notes were taken during the interview, which lasted approximately 25 minutes (the minimum duration was 20 min, and the maximum duration was 35 min).

### Data Analysis

#### Quantitative Evaluation

SPSS software (version 26.0; IBM Corp) was used for all statistical analyses. Descriptive statistics were used to describe all variables; frequency and mean with SD were reported.

#### Qualitative Analysis

Participant interviews were digitally recorded, transcribed verbatim in their language (Arabic), and coded using the MAXQDA qualitative analysis software (version 12.0; VERBI GmbH) [56]. Although the participants could read and speak English, the interviews were conducted in Arabic because people feel more confident speaking their first language. A thematic framework analysis was performed to identify the key themes within the data [57,58]. The thematic framework was developed through a combination of inductive and deductive approaches. A framework analysis was selected for its effectiveness as a tool for qualitative content analysis. This method provides a systematic approach to organizing and mapping data [58]. It is valuable for analyzing interview data, comparing themes within and between cases, and managing large datasets by offering a structured data summary [58]. Inductive methods involved identifying and categorizing emerging ideas into preliminary themes, while deductive methods mapped these themes based on social cognitive theory [21]. Two researchers (GA and JZA) independently analyzed and explored the transcript data, taking notes on preexisting and emerging themes. These notes were used to create a coding framework applied to the transcript data within MAXQDA. The data within each theme were then thoroughly examined and summarized, including differences between participants, to create summaries of each theme and identify similarities and differences between themes. After discussions with EAW and MSH regarding the final analysis stage results, GA implemented some adjustments. In this study, quantitative and qualitative data were analyzed separately [59]. In the final interpretation of the results, the quantitative data complemented the qualitative data [59,60]; for example, in the case of missing data, interviews may show why one point of measurement has more missing data than another. This study used an integration matrix (Multimedia Appendix 3) to compare data obtained from different methods.

### Ethical Considerations

This study received approval from the University of Sheffield’s ethics committee (053461) and Princess Nourah bint Abdul Rahman University (23-0453). The study details were communicated to participants through information sheets sent to all eligible individuals by email (participants’ email addresses were obtained from the previous study). A researcher (GA) contacted eligible participants to provide additional information about the study (eg, study protocol and duration) and obtain electronic informed consent. The study data were anonymized, deidentified, and stored securely in a password-protected file in a secure filestore. No compensation was provided to participants for taking part in the study; however, they were reimbursed the cost of the Noom app.

## Results

### Recruitment and Retention Rate

Fourteen individuals with high blood pressure took part in the feasibility study. Their average age was 43 (SD 8.11; range 35-65) years. Of the 14 participants, 8 (57%) were male, and 10 (71%) held a bachelor’s degree. Most of the participants (9/14, 64%) had systolic blood pressure between 140 mm Hg and 159 mm Hg and diastolic blood pressure between 90 mm Hg and 99 mm Hg. The demographics of the participants are illustrated in Table 1. All 14 participants completed the baseline questionnaire. Of the 14 participants, 2 (14%) withdrew during the trial (n=1, 50% in week 2 and n=1, 50% in week 5). Both participants voluntarily reported that they withdrew because they found the research, especially the required dietary log, too burdensome. Of the remaining 12 participants, 9 (75%) completed all data collection points.

**Table 1:** Baseline demographic characteristics of participants with high blood pressure recruited to the study (n=14).

Characteristics		Values
Age (y), mean (SD; range)		43 (8.11; 35-65)
**Sex, n (%)**		
	Male	8 (57)
	Female	6 (43)
**Marital status, n (%)**		
	Single	2 (14)
	Married	12 (86)
**Education, n (%)**		
	High school diploma	1 (7)
	Bachelor’s degree	10 (71)
	Master’s degree or above	3 (21)
BMI (kg/m^2^), mean (SD)		30.7 (5.54)
Participants with BMI 25-30 kg/m^2^, n (%)		6 (43)
Participants with BMI 30-39.9 kg/m^2^, n (%)		8 (57)
Participants with blood pressure readings from 130/85 mm Hg to 139/89 mm Hg, n (%)		5 (36)
Participants with blood pressure readings from 140/90 mm Hg to 159/99 mm Hg, n (%)		9 (64)
Blood pressure medications (self-reported), n (%)		9 (64)
Users of nutrition smartphone apps, n (%)		2 (14)

### Engagement With the Noom App

Table 2 summarizes the participants’ engagement during weeks 4 and 8. All participants logged their food using the app. Most of the participants (8/13, 62%) logged their food for 3 to 5 days a week, with the frequency of food logging increasing with longer periods spent using the app. The meal record of each user was used to measure their engagement with the app over the 2 months. During the first month, of the 13 participants, 1 (8%) spent 31 to 45 minutes per day on the app, 6 (46%) spent 16 to 30 minutes per day, and 6 (46%) spent 1 to 15 minutes per day. In the following month, of the 12 participants, 1 (8%) spent approximately 30 minutes, while the sessions of most users (n=11, 92%) decreased to 1 to 15 minutes. During the initial month, most of the participants (9/13, 69%) logged their meals at the end of the day, although the frequency of this practice slightly decreased over time, snacks being the foods that participants most frequently forgot to record.

**Table 2:** Self-reported frequency of engagement and food logging with the Noom app assessed at follow-up appointments at week 4 and week 8 using a questionnaire.

Engagement measure^a^		Weeks 1-4 (n=13), n (%)	Weeks 5-8 (n=12), n (%)
**Food logging on the Noom app (d/wk)**			
	7	3 (23)	5 (42)
	3-5	8 (62)	6 (50)
	1	2 (15)	1 (8)
**App use (min/d)**			
	31-45	1 (8)	0 (0)
	16-30	6 (46)	1 (8)
	1-15	6 (46)	11 (92)
**Daily meal recorded**			
	1	1 (8)	1 (8)
	Most	9 (69)	6 (50)
	All	3 (23)	5 (42)
**Time of day for food logging**			
	At the end of the day	9 (69)	6 (50)
	Meal by meal	4 (31)	6 (50)
**Meals not recorded**			
	Breakfast	3 (23)	1 (8)
	Lunch	2 (15)	2 (17)
	Dinner	3 (23)	1 (8)
	Snacks	5 (38)	8 (67)

^a^Participants were asked to report the frequency of their engagement with regard to food logging with the Noom app over the previous 4 weeks. This included documenting the frequency of logging, such as the number of days, minutes, daily meals recorded; meals not recorded; and the time of day for food logging.

### DASH Diet Adherence and Self-Efficacy

As shown in Table 3, over the 8-week intervention period, participants tended to increase their DASH diet score. Participants also reported decreased total fat, saturated fat, cholesterol, and sodium levels and increased calcium, magnesium, and protein intake. In addition, the self-efficacy score, measured out of 30, increased, indicating higher self-efficacy. The participants’ mean self-efficacy score was 18 (SD 4.7) at baseline (n=14) and 20 (SD 6.3) at the 8-week follow-up (12/14, 86%).

**Table 3:** Change in DASH (Dietary Approaches to Stop Hypertension) adherence score and DASH score components among participants with high blood pressure from baseline to follow-up.

DASH score components	Mellen DASH Index^a^ [46]		Baseline (n=14), mean (SD)	At 8-week follow-up (n=12), mean (SD)
	DASH score target	Intermediate target		
DASH score (out of 9)	—^b^	—	3.4 (1.4)	4.3 (1.1)
Total fat, energy (% total kcal)	27	32	35.9 (8.1)	28.2 (6.5)
Saturated fat, energy (% total kcal)	6	11	11.0 (4.1)	9.3 (5.4)
Protein, energy (% total kcal)	18	16.5	17.9 (3.3)	21.3 (3.4)
Cholesterol (mg/1000 kcal/d)	71.4	107.1	245.0 (206.0)	230.0 (167.0)
Fiber (g/1000 kcal/d)	14.8	9.5	13.1 (5.7)	12 (5.6)
Magnesium (mg/1000 kcal/d)	238	158	138.0 (78.0)	146.0 (66.0)
Calcium (mg/1000 kcal/d)	590	402	360.0 (185.0)	506.0 (130.0)
Potassium (mg/1000 kcal/d)	2238	1534	1632.0 (705.0)	1489.5 (474.0)
Sodium (mg/1000 kcal/d)	1143	1286	1503.0 (772.0)	1181.0 (637.0)

^a^Each participant receives 1 point for meeting the target, 0.5 points for meeting the intermediate target, and 0 points for not meeting either target.

^b^Not applicable.

### SUS Questionnaire Responses

On the basis of the responses to the SUS questionnaire, it was found that all of the respondents had a positive experience with the Noom app (Table 4), with an overall mean SUS score of 73.33 (SD 8.07; range 62.50-87.50), indicating that the Noom app is acceptable. The question with the most positive answer revealed that users found the app to be consistent and not cumbersome. However, 1 question received a mix of negative and neutral responses, where users felt they needed to learn a lot before using the Noom app.

**Table 4:** Participant responses to the System Usability Scale (SUS) questionnaire and total SUS score after 8 weeks of using the Noom app (n=12)^a^.

SUS questions	Positive responses, n (%)	Neutral responses, n (%)	Negative responses, n (%)
I think that I would like to use the Noom app frequently.	11 (92)	1 (8)	0 (0)
I found the Noom app unnecessarily complex.	11 (92)	1 (8)	0 (0)
I thought the Noom app was easy to use.	10 (83)	2 (17)	0 (0)
I think that I would need the support of a technical person to be able to use the Noom app.	9 (75)	3 (25)	0 (0)
I found the various functions in the Noom app were well integrated.	9 (75)	3 (25)	0 (0)
I thought there was too much inconsistency in the Noom app.	12 (100)	0 (0)	0 (0)
I would imagine that most people would learn to use the Noom app very quickly.	7 (58)	3 (25)	2 (17)
I found the Noom app very cumbersome (inconvenient) to use.	12 (100)	0 (0)	0 (0)
I felt very confident using the Noom app.	10 (83)	2 (17)	0 (0)
I needed to learn a lot of things before I could get going with the Noom app.	6 (50)	3 (25)	3 (25)

^a^Overall SUS score: mean 73.22 (SD 8.07).

### Qualitative Results

#### Overview

The one-on-one interviews were conducted with 9 (75%) of the 12 participants who completed the study. The data analysis revealed four overarching descriptive themes concerning the Noom app’s use over the previous 8 weeks: (1) acceptance, (2) app usability, (3) technical issues, and (4) suggestions for improvement. The thematic framework analysis is presented in Multimedia Appendix 4.

#### Acceptance

The Noom app was generally well accepted and liked among the participants who completed the study. Most of the participants generally found the Noom app easy to use, and as a result of consistently tracking their behavior, most were able to re-evaluate their dietary choices and break old habits (as shown in the quantitative data, of the 12 participants, 8 (67%) had improved their DASH diet scores):

Generally, I am a busy person; when I started using the app and noticed the positive feedback, it satisfied me because I was trying to modify my dietary behavior.

Participant 4, male, aged 35 years, bachelor’s degree

[The Noom app] helped me change my dietary behavior. Every morning, I was feeling that I needed to use the application.

Participant 6, male, aged 49 years, bachelor’s degree

Most of the participants experienced positive changes in their behavior due to self-monitoring and receiving regular feedback through the app. The app color coded positive and negative energy balances—green for positive, yellow for neutral, and orange for negative—which helped them become more aware of their dietary habits and encouraged them to reflect on their behaviors. Furthermore, the feedback allowed them to make improvements, eventually increasing their self-efficacy and DASH score, as evidenced by the quantitative data:

Upon the appearance of green color, I felt that I reached my goal. And by receiving red color I felt this was a compelling reason to stop eating unhealthy food, especially upon logging your meals on time.

Participant 8, female, aged 45 years, bachelor’s degree

Many of the female participants were able to positively impact their children’s eating habits by incorporating more fruits and vegetables into their diets, serving healthy snacks, and having dinner earlier (one of the participants even suggested that her friend try using the Noom app):

My awareness increased, and I tried to modify my children’s behaviors; I became keen on increasing the amount of fruit and vegetable intake, and we had our dinner early.

Participant 9, female, aged 44 years, bachelor’s degree

There were mixed opinions regarding the reminders (sent through the app) for logging food and reading articles. Some found these messages helpful and promptly acted on them, while others ignored them but still appreciated receiving them because they helped them log the meals missing from their record. However, some of the participants did not find the reminders helpful, and they disabled the notifications because they received them at inconvenient times and found them annoying:

Because I was busy during the day, I turned off all notifications, except the last notification of the day, to remind me to log my meals.

Participant 4, male, aged 35 years, bachelor’s degree

The notifications are useful, especially when the person is lazy or forgetful. They remind the person and help him adhere to the application’s instructions.

Participant 6, male, aged 49 years, bachelor’s degree

Most of the participants reported that effective app use required self-motivation to change behaviors. They were self-motivated to continue using the app due to positive outcomes, health concerns, and daily goals such as calorie limits. Moreover, some reported that the app provided daily motivational messages that encouraged them to continue using it:

The motivation is that I became able to control food quantity, especially since I am at risk of developing diabetes.

Participant 5, male, aged 41 years, master’s degree

Motivating messages and easiness of application usage such as when you forget to log my meal, I received a message saying that don’t worry you still have a chance and time has not finished yet. I also remember that at the beginning of the course, I received a message saying, “Starting is very difficult, but ends are very pleasing.” These phrases give me the desire and motivation to continue.

Participant 7, female, aged 40 years, bachelor’s degree

Participants had differing opinions about using the app when they were busy or on holiday. The app benefited some because it allowed them to manage their food intake during the holidays, specifically during Eid ul-Adha (Festival of Sacrifice), while others did not use the app during this time (this reason could explain the differences in app engagement):

Eid ul-Adha significantly influenced us, during which we ate lots of meat and sweets, but the application helped me to control the food quantity; I ate only one piece of meat instead of two.

Participant 2, male, aged 43 years, bachelor’s degree

During the holidays, I use the application notifications to remind me to adhere to eating healthy food, but I do not log my meals onto the application due to difficulty in logging meals every day.

Participant 6, male, aged 49 years, bachelor’s degree

Participants felt that they benefited both psychologically and behaviorally from the app’s educational component. Most of the participants felt that the Noom app provided them with information to increase their confidence in selecting healthier food, monitor their dietary intake, and incentivize eating healthy food:

The educational information is beneficial, especially psychologically, in controlling meal selection and diplomatically behaving with your family or friends regarding choosing healthy food. The application directs you to the best choice, showing its health advantages. The application also gives you new information about the quality, quantity, and time of food and method of eating, for example, how you can chew.

Participant 1 male, aged 65 years, bachelor’s degree

However, all participants expressed their dissatisfaction with some of the Noom app’s suggestions and language. Some of the app’s recommendations cater more to Westerners and do not consider Arab and Muslim cultural differences; for instance, some of the users found it odd that the app suggested limiting alcohol consumption to only 1 serving. Furthermore, the app incorporates American slang (eg, “listen to your gut,” “take the plunge,” and “veggies”), which can be confusing for some users who are not familiar with the language:

The application has a simple defect: it talks about American culture. No doubt, there is a comprehensive food database that contains Arab and international meals from other countries. But it deals with me as an American and gives me advice appropriate to their culture, such as eating pork and drinking alcohol, and practicing American sports, such as dancing.

Participant 1, male, aged 65 years, bachelor’s degree

#### App Usability

Most of the participants reported that the Noom database contained a variety of foods and was easy to use, convenient, and valuable. However, participants reported difficulties in identifying certain foods due to a lack of local options on the app, such as local restaurant foods and traditional cuisine. Mixed dishes, such as *kabsah* (a traditional Saudi Arabian dish with rice and meat), and homemade foods also posed challenges for some of the participants. Regarding the option for users to add their foods to the database, only a few of the participants found this helpful. Some of the users expressed concerns about the accuracy of food information in the database after adding their foods:

The food database needs to be more accurate, but it is better than nothing [for dietary self-monitoring].

Participant 1, male, aged 65 years, bachelor’s degree

There is an advantage which satisfied me: the ability of the user to enter his meals and to be available to others, but my question is there someone who reviews our meals entering because it is possible that our estimation of elements is not accurate, therefore the database is not accurate.

Participant 5, male, aged 41 years, master’s degree

Some of the participants reported that they were unable to incorporate the app into their daily routine quickly, which was supported by their SUS score for the question “I would imagine that most people would learn to use the Noom app very quickly”:

In the beginning, I was upset, and the application didn’t satisfy me, because I searched in a database which required further time and effort but after a period of time, I have adapted to it.

Participant 4, male, aged 35 years, bachelor’s degree

#### Technical Issues

Participants reported technical errors, including the app frequently freezing or responding slowly; this happened to 4 (44%) of the 9 participants. They solved this issue by uninstalling and reinstalling the app:

Technical problems causing the suspension of the application once or twice a week and I have to uninstall the application and reinstall it.

Participant 1, male, aged 65 years, bachelor’s degree

When participants encountered technical problems, they generally did not contact the primary researcher. These problems were usually only discovered during the interviews. Some of the participants hesitated to trouble the researchers with technical issues stemming from their slow internet connections. By contrast, a few of the participants preferred to contact the Noom app support team to address the technical problems because they believed that the support staff were accountable for such matters:

There is a simple technical problem that the application didn’t respond may be due to internet weakness.

Participant 6, male, aged 49 years, bachelor’s degree

*I contacted Noomapp support about this issue, and they replied they were aware of the issue and were working to resolve it.* [Participant 5, male, aged 41 years, master’s degree]

#### Suggested Improvements

There were some suggestions to improve the Noom app. These suggestions included developing an Arabic version, reviewing its content, and deleting any recommendations unsuitable for Arab and Muslim populations:

Please develop the application [the Arabic version] to reflect our Arabic culture. It would be best to remove any inappropriate content, such as references to alcohol consumption, to make it more suitable for the Arab user.

Participant 6, male, aged 49 years, bachelor’s degree

To be honest, I wish there was an option like the Arabic language in the language settings because not everyone can read and understand English.

Participant 7, female, aged 40 years, bachelor’s degree

Furthermore, participants believed that adding some traditional Saudi Arabian and Arab cuisine would be a useful addition because people from all backgrounds and ages would benefit from this, making the app more appealing to users from these regions. Participants also suggested that it should have offline functionality and that subscription prices should be reduced. These changes would improve the app’s accessibility and usefulness for all users:

*I want an Arabic version of the Noomapplication that is in line with the Arab and Middle Eastern cultures, also its price is expensive.* [Participant 4, male, aged 35 years, bachelor’s degree]

To add some traditional Arab cuisine to its database and to work without internet, because sometimes I have no internet and find it difficult to log meals after returning home.

Participant 2, male, aged 43 years, bachelor’s degree

A final suggestion was to find a method to make logging food easier (although the Noom app a barcode scanner for identifying food, it seems unable to recognize some of the local Saudi Arabian products):

Sometimes I feel lazy or forget what I ate for breakfast and lunch, so I did not log my meals. I would like it if there was an easier way to log my meals on time.

Participant 5, male, aged 41 years, master’s degree

## Discussion

### Principal Findings

#### Overview

This study examined the feasibility and acceptability of using the Noom app to assist people with high blood pressure to improve their dietary behaviors. After an 8-week intervention, this study found it feasible and acceptable to use a commercially available app to support DASH diet self-management among people with high blood pressure in Saudi Arabia. Moreover, there was a positive trend toward an increase in DASH scores and self-efficacy, but these results were not statistically significant.

#### The Intervention’s Feasibility and Acceptability

The study recruited 14 people with high blood pressure. The participants who took part in the interview (9/14, 64%) expressed positive opinions about the intervention. Many of the participants reported significant benefits from the intervention in terms of their motivation to change their dietary behavior and their psychological or physical well-being. Most of the participants found the Noom app easy to use, and most had no difficulties integrating it into their daily routines.

However, of the 14 participants, 2 (14%) withdrew from the study; furthermore, of the 12 participants who completed the study, 3 (25%) did not take part in the interview. Some found the app overwhelming when monitoring their diet. This feedback was based on participants’ reasons for withdrawal. The findings indicate that dietary smartphone apps may be more effective for individuals with high motivation and willpower. Consequently, an intervention based on a dietary smartphone app may not be beneficial for those who are less motivated and less willing. Similarly, a previous study assessed the use of the MyFitnessPal app for weight loss [61]. It concluded that clinicians may not necessarily recommend the app to every patient who is overweight and has a smartphone unless they are motivated to lose weight and track their calorie intake. For these patients, the app could be a helpful tool [61]. However, our study’s findings should be interpreted cautiously due to the small sample size and short study period.

Dietary smartphone apps have been found to be a promising intervention for supporting DASH diet self-management [30]. Our study found that the Noom smartphone app seemed to improve adherence to the DASH diet at 8 weeks. These findings are consistent with a recent randomized controlled trial conducted in Iran, which revealed that a smartphone app improved adherence to the DASH diet plan [62], highlighted by a clinically meaningful difference in DASH indexes between the two groups at the end of the study. Nonetheless, the results did not reach statistical significance. Most of the participants in our study suggested that personalized feedback, motivational reminders, and knowledge were essential to increase participants’ DASH adherence and app engagement. This aligns with the principles of behavior change theories, which emphasize the importance of goal setting, feedback, and knowledge for successful self-management and behavioral control [63,64].

Most of the participants (8/13, 62%) in this 4-week study tracked their diet for 3 to 5 days a week. During the second month, the number of days the participants used the app generally increased, while the time spent per day on it decreased. The results of this study contradict those of the study by Laing et al [61], who found that the use of MyFitnessPal, a popular app for diet tracking, decreased considerably after the first month [61]. In our study, it is possible that the motivational reminders and feedback provided by Noom, the need to log meals missing from the record, additional meals (the meals that are not found in the food database), and saving favorite meals encouraged the participants to maintain a higher frequency of engagement. In addition, a possible explanation for the decrease in time spent per day engaging with the app is its time-saving features, such as the option to save favorite meals and foods [65]. Ziesemer et al [66] reported that fixed daily reminders and logging meals missing from the record could increase the awareness and number of logged eating events in the short term. However, the long-term effects of fixed reminders remain unknown. Moreover, most of the participants acknowledged that perceived missed events were likelier to be snacks than main meals because they logged their food at the end of the day, forgot having had snacks, were busy, or had no Wi-Fi access. Similarly, the study by Ziesemer et al [66] found that snacks were more likely to go unrecorded. The most common reasons were obstacles related to devices (eg, the absence of a device at hand), multitasking, or situational barriers [66]. Further research is needed to identify events that are likelier to go unrecorded and to determine the reasons for such omissions.

Moreover, studies show that individuals who practice self-monitoring tend to experience greater success in changing their health behaviors [20,67]. The Noom app includes information on calorie balance, which helped participants confidently estimate the calorie content of foods. Furthermore, participants’ feelings toward certain types of foods were influenced by Noom. This led to heightened awareness among the participants, increasing their consumption of fruits and vegetables and decreasing their consumption of unhealthy snacks, fast food, soft drinks, and high-fat foods such as red meat. Some of the participants also found the app helpful in regulating their food intake. Engaging in self-monitoring behavior can help users gain knowledge about which foods are beneficial or detrimental to their diet, ultimately improving their ability to achieve their goals [20].

In addition, after using the Noom app, some of the mothers among the participants decided to increase the amount of fruits and vegetables they served their children. This positive influence of the Noom app aligns with the results of a systematic review [68] highlighting the effectiveness of digital interventions in promoting nutrition among parents and improving outcomes for both parents and children.

Another factor that positively influenced the acceptance of the Noom app was the positive feedback and motivational information provided in the educational information. After using the app, participants reported feeling more motivated and capable of improving their dietary habits. They also experienced an increase in their ability to set and achieve dietary goals. Bozorgi et al [69] demonstrated that using a mobile app–based education intervention improved adherence to low-fat and low-salt diets among patients with high blood pressure. Participants’ self-efficacy level was also increased during our study. Our findings are consistent with prior research showing that diet apps can improve self-efficacy or improve individuals’ belief that they can engage in healthy eating behaviors [70]. The findings of our study are theoretically supported by social change theory, which argues that individuals’ confidence that they can consume healthy food daily, despite challenges, is often a major determinant of their ability to adhere to a healthy diet [21]. From a psychological perspective, positive or negative consequences for the individual’s health are essential factors influencing behavior [71]. Therefore, when developing nutrition apps, it is crucial to avoid fostering feelings of shame by presenting information in a positive manner.

Although the Noom app was considered an acceptable tool, some of its educational content proved unsuitable for individuals with elevated blood pressure in Saudi Arabia. Participants refused to adopt some of the app’s suggestions that did not align with their cultural beliefs; for instance, the app recommended having a nightcap before sleep, which was not well received. Empirical studies have shown that health and diet promotion initiatives that consider culture, ethnicity, and language are more effective in motivating individuals to adopt healthy behaviors [72]. Therefore, it is crucial that personalized nutrition advice and messaging are provided, along with tailored recommendations for affordable and accessible meals and recipes that align with an individual’s daily diet [73]. As König et al [71] pointed out, besides technological factors, the characteristics of a potential user, the interaction between the user and the technology, and the social environment also play a role in the use of nutrition apps. Alzahrani et al [74] developed an app called Sehhaty Wa Daghty (Arabic for “health and blood pressure”) to cater to the cultural and social norms of Saudi Arabia, the motivational needs of male and female Saudi Arabian citizens, and their hypertension management needs. This app allows for easy tracking of blood pressure, physical activity, and a healthy diet, and it provides reminders for medication intake and water consumption. Most of the participants found the Sehhaty Wa Daghty app easy to use and acceptable [74]. There are notable differences between this intervention and ours. The research team developed an app for their study [74] rather than choosing one that was commercially available. Consequently, the app underwent prototype testing, and the researchers had to consider many user design aspects more appropriate for the Saudi Arabian population. Nonetheless, it is worth noting that the use of the Sehhaty Wa Daghty app was limited to the study population, while the Noom app is accessible to the general public. Alzahrani et al [74] discuss valuable insights on the development of a smartphone app for self-management of hypertension among Saudi Arabian patients. They emphasize the significance of considering cultural and social norms when creating digital health programs in Saudi Arabia. Our findings add to this evidence and suggest that commercial dietary smartphone apps have the potential to be used effectively among the Saudi Arabian population.

The accuracy of the food database is crucial to the Noom app’s user acceptance. Although the app offers a comprehensive food database—including data from government sources, such as the US Department of Agriculture’s food database; international restaurant chains; and some Arab cuisines—it lacks many traditional Saudi Arabian cuisines, which affects food tracking. Many Saudi Arabian participants reported that food data entry was complex due to the need to estimate portion sizes as well as calorie, fat, and protein content. They were also concerned about the food database’s accuracy and reliability. A previous systematic review has demonstrated that issues related to the usability of food intake tracking can affect users’ willingness to record their food intake [71]. The systematic review also emphasized that nutrition apps that allow users to enter data are prone to human error; consequently, nutritional values are likely incorrect, and their sources could be uncertain [71]. Therefore, using simpler input methods, such as using household items to indicate portion sizes or photo-based food recording, can alleviate the burden on users [71]. To increase users’ trust, it is crucial to create opportunities to enhance transparency regarding food data sources if human error cannot be avoided completely [71].

The Noom app worked well most of the time. Nevertheless, the most challenging technical issues were freezing and slow response times. The support team assured users that they were working on a solution. These technical issues, as well as app malfunctions, can lead to user disengagement [71]. Therefore, these issues must be resolved before conducting future studies to ensure that the app can be widely adopted.

Most of the participants followed the DASH diet but could not fully adhere to it. Steinberg et al [47] found that lower-intensity approaches that focused solely on dietary behavior had only a slight impact on adherence to the DASH diet. Moreover, it has been challenging to achieve complete adherence even in DASH trials that have included intensive behavioral interventions [75]. In the PREMIER trial [76,77], a comprehensive and multicomponent behavioral intervention was used to improve the adoption of the DASH diet. The intervention consisted of group meetings and frequent face-to-face counseling sessions with a registered dietitian. The intervention included sodium reduction, increased physical activity, and weight loss [76]. Although the intervention effectively improved DASH diet adherence, it did not lead to full adoption. Although the DASH recommendations are comprehensive, their full adoption may be challenging when the focus is on multiple behaviors simultaneously. A previous study indicated that although full adherence was optimal, partial adherence to the DASH diet could lower blood pressure [47].

### Recommendations

Although the Noom app may be suitable for individuals with high blood pressure in Saudi Arabia, not all participants were satisfied with some of its content. Our results suggested that an Arabic version of the Noom app that is more suitable for Arab users needs to be developed; content that does not align with Saudi Arabian cultural values needs to be removed.

Our results suggest that the food-tracking method should be simplified to reduce the burden of text-based data input. The Noom app includes a barcode scanner; however, some of the participants could not find their foods, or some foods did not come with barcodes. To promote continuous use of the app, participants in this study suggested incorporating voice technology. Previous research has indicated that logging diets using voice is more effective and more user-friendly than text-based methods [78]. Furthermore, implementing an offline mode so that users can monitor their food intake and access the app even without an internet connection is recommended [79].

### Strengths and Limitations

One strength of the feasibility study was that it evaluated the Noom app over 2 months in individuals who experienced high blood pressure in real-life conditions. To our knowledge, no previous research has used a commercial dietary app to support people with high blood pressure in Saudi Arabia.

Several limitations of this study should be noted. First, similar to previous dietary studies, the results of this study may have been limited by the accuracy of the data collected, which may have been influenced by recall and response biases [80,81]. Some of the participants may have overestimated or underestimated their dietary intake because they knew that their diet data would be collected. In addition, because all measurements were self-reported, inaccuracies and biases may have been introduced. Second, the study population consisted of well-educated individuals who read and spoke English and were familiar with technology. Therefore, further research is needed to determine whether these findings are generalizable to less highly educated people. Finally, because some of the participants dropped out or did not take part in the interviews, we may have missed some valuable insights regarding the usability and acceptance of the intervention.

### Conclusions

The results of this feasibility study showed that using dietary smartphone apps among people with high blood pressure in Saudi Arabia is both feasible and acceptable. There was an apparent improvement in DASH diet adherence and self-efficacy, supporting the need for a larger trial. The results indicate that the food-tracking method should be simplified (eg, by incorporating voice technology for food tracking). Furthermore, this study provided insights into the app’s educational content, which was not always suitable for Saudi Arabian users, and highlighted the need for a culturally appropriate Arabic version of the app before conducting a full trial.

## Appendix

Multimedia Appendix 1 CONSORT (Consolidated Standards of Reporting Trials) 2010 checklist.

Multimedia Appendix 2 Interview guide.

Multimedia Appendix 3 Integration matrix.

Multimedia Appendix 4 Thematic framework analysis.

## Abbreviations

CONSORT: Consolidated Standards of Reporting Trials
CVD: cardiovascular disease
DASH: Dietary Approaches to Stop Hypertension
SUS: System Usability Scale

## Data Availability

The datasets generated and analyzed during this study are available from the corresponding author on reasonable request.
