# Supplementary material for: An Investigation of the Feasibility and Acceptability of Using a Commercial DASH (Dietary Approaches to Stop Hypertension) App in People With High Blood Pressure: Mixed Methods Study
Source: JMIR Form Res. 2024 Nov 19;8:e60037. doi: 10.2196/60037 (PMC11615541; doi:10.2196/60037)
Supplement: Multimedia Appendix 2 [file formative_v8i1e60037_app2.docx]

**The interview guide**

NOOM has been available to you for eight weeks, and you have had access to its various features. You have received notifications to log your food, had the opportunity to set goals, view your dietary habits, and receive feedback. Could you share your experience with these features with us? Due to this, I will ask questions about it in today's interview. Do you have any questions before we begin? Can you consent to participating in the interview and recording it (audio)?

1. Can you tell me about your experience using Noom?
2. Can you tell me about any aspects of Noom you liked?
3. PROMPTS:

- Ease of use and convenience
- Design (color, information, and language)

1. Can you tell me about any aspects of Noom you disliked?

- Technical issue.
- Record your food every day.
- Reminder to log your food or to read articles.

1. How did using Noom app for tracking your food/drink impact you? Why / why not?

**PROMPTS:**

- **Improve your dietary habits.**

1. How do you think it changed how you thought about your food choices, if at all?
2. Did using the app change your time thinking about your food choices?
3. Did you need to use Google Translation or research to understand any topic?
4. Did anything happen in your life that made using the app challenging? For example, travel, holiday, or Eid?
5. Do you have any questions?

***Thank you for participating in this interview!***
