# Supplementary material for: An Investigation of the Feasibility and Acceptability of Using a Commercial DASH (Dietary Approaches to Stop Hypertension) App in People With High Blood Pressure: Mixed Methods Study
Source: JMIR Form Res. 2024 Nov 19;8:e60037. doi: 10.2196/60037 (PMC11615541; doi:10.2196/60037)
Supplement: Multimedia Appendix 3 [file formative_v8i1e60037_app3.docx]

| **Research questions** | **Engagement with Diet Tracking *(QUAN)*** | **DASH Diet Adherence *(QUAN)*** | **DASH Diet Self-Efficacy *(QUAN)*** | **system usability scale *(QUAN)*** | **Interviews *(QUAL)*** | **Comments to integration**  ***(QUAN+QUAL)*** |
| --- | --- | --- | --- | --- | --- | --- |
| Is it feasible to use the Noom app to change dietary behaviour in hypertension patients and their self-efficacy?  Is the Noom app acceptable to people with raised blood pressure? | Most participants (61%) logged their food 3-5 days a week and spent an average of 15 minutes using the app. | Participants had a small increase in DASH score over 8 weeks | Participants self-efficacy had slightly increased over 8 weeks | The overall mean SUS score was 73.7 (8.07); it indicates that the Noom app is acceptable.  In response to question 10, half of the users indicated that they would need to learn a lot before using the Noom app. | Theme: acceptance  Most users found the app very user-friendly and had no trouble making it a part of their daily routine. Moreover, the app is an excellent motivator for users to maintain healthy dietary habits. The app also offers various features that enable users to interact with it effectively and provide helpful feedback, motivational messages, and reminders to keep them on track with their dietary goals. However, all participants were not satisfied with some of the app suggestions, which did not impact their engagement with the app.  Using the app during the holiday received mixed reviews from participants. While some found it helpful in controlling their dietary behaviour, others found it challenging.  **Theme: app usability**  Despite the easy app, some participants faced challenges logging complex food and traditional cuisine—also some concerns about the food database accuracy. | The questionnaires and interviews are very **similar**, but the interviews provide more details about the reasons why users are satisfied with this application and why not.  In addition, the difference in opinions among participants regarding using the app during holidays and usability issues could account for their varying levels of engagement with the app.  **In the interview**, the participants shared that they needed to acquire a lot of knowledge before using the app, as they encountered difficulties while trying to log complex meals. |
| Are there any technical problems with the Noom app? |  |  |  | Three participants who responded to the question believed they would require technical assistance to use the Noom app. | **Theme: technical issue.** During the interview, we explored that the Noom app frequently freezes twice weekly or responds slowly; this happened to four participants. They solved this issue by uninstalling and reinstalling the app. Also, they contacted the Noom team support. | The questionnaires and interviews are very **similar**, but participants provided more details about the technical issues they faced and how they solved them during the interview. |
| Should any changes be made to increase the feasibility of using the Noom app? |  |  |  |  | **Theme: Suggested Improvements** There were some suggestions to increase the feasibility of using the Noom app, including translating it into Arabic, reviewing its content, and deleting any recommendations unsuitable for the Arab and Muslim populations. | Nothing to integrate |
