# Supplementary material for: An Investigation of the Feasibility and Acceptability of Using a Commercial DASH (Dietary Approaches to Stop Hypertension) App in People With High Blood Pressure: Mixed Methods Study
Source: JMIR Form Res. 2024 Nov 19;8:e60037. doi: 10.2196/60037 (PMC11615541; doi:10.2196/60037)
Supplement: Multimedia Appendix 4 [file formative_v8i1e60037_app4.docx]

| **Theme** | **Sub-theme** | **Initial codes** |
| --- | --- | --- |
| *Acceptance* ***^a^*** | *Using the app and monitoring* | Modify dietary behaviour, break old habits, integrate the app into daily routines, self-monitoring and feedback, |
|  | Encouraging a culture of healthy eating | Recommend the app to friends, increase vegetable and fruit serving sizes for their children, eat healthy snacks, having dinner earlier. |
|  | *App functions* | **1) Push notifications**, helpful, inconvenient times, annoying.  **2)Daily goals** such as calorie limits.  **3)motivational messages**  **4)educational components,** selecting healthier food, monitoring their dietary intake  5) **Dietary self-monitoring and feedback:** regulating food intake, increasing awareness, and gaining knowledge. |
|  | App disengagement | busy or holiday |
|  | dissatisfaction | suggestions and language, including limiting alcohol consumption, pork and incorporating American slang |
| *app usability* | *How easy to use* | The food database is convenient and valuable. |
|  | *Challenges* | missing local options, mixed dishes, adding foods to the database, accuracy of food information, and integrating the app into their daily routine quickly. |
| *technical issues* | *freezes or responds slowly* | uninstalling and reinstalling the app, slow internet connections, and Noom app support team |
| suggestions for improvement | List of suggestions | Translating it into Arabic, reviewing its content, adding traditional Saudi and Arab cuisine, using it offline, decreasing the cost, and simplifying the method of logging food. |

**a** A priori themes are indicated in italics
